# Reappraising Sexual Coevolution and the Sex Roles

**DOI:** 10.1371/journal.pbio.1000255

**Published:** 2009-12-08

**Authors:** Russell Bonduriansky

**Affiliations:** 1 Evolution and Ecology Research Centre, University of New South Wales, Sydney, New South Wales, Australia; 2 School of Biological, Earth, and Environmental Sciences, University of New South Wales, Sydney, New South Wales, Australia

## Abstract

Over recent decades, new ideas have radically altered how we see sex and reproduction. The implications of these ideas are still being explored, yielding intriguing discoveries.

The history of evolutionary biology illustrates how theory shapes what we see and don't see in nature. Over the past 30 years, theoretical reappraisals in two areas of evolutionary research—sexual coevolution and the sex roles—have challenged longstanding ideas and yielded rich harvests of startling observations. This process continues apace.

## The Legacy of Darwin and Bateman

Charles Darwin [1],[2] handed us a picture of competition as a constructive process responsible for adaptation to the environment, as well as the evolution of striking sexual displays and weapons. Darwin believed that sexual competition promotes adaptation by helping to weed out poor-quality males from the breeding pool and bringing about assortative pairing between high-quality males and females.

“…the largest number of vigorous offspring will be reared from the pairing of the strongest and best-armed males, victorious in contests over other males, with the most vigorous and best-nourished females…” [2].

Darwin also bequeathed to us his idea of the “typical” roles of the sexes: eager males competing for the affections of choosy females. Darwin was careful to point out that “reversed” sex roles occur in some species, noting the “much rarer case of the males selecting particular females” [2], but these were exceptions to the rule.

In 1948, Angus Bateman [3] furnished an apparent experimental corroboration of Darwin's view of the sex roles through experiments with *Drosophila melanogaster*. Bateman showed that male fitness was highly variable and increased with each mating, whereas female fitness was less variable and appeared to increase little (or even decline) with additional matings. Bateman believed that the fundamental difference between sexes in gamete size (i.e., anisogamy: males' production of numerous, tiny sperm and females' production of few, large, energetically costly eggs) ultimately explained why “in unisexual organisms there is nearly always a combination of an undiscriminating eagerness in the males and a discriminating passivity in the females.”

The Darwin-Bateman theory of the sex roles, extended by Robert Trivers [4], formed the theoretical bedrock of the emerging field of behavioural ecology. When the study of mating systems and sexual selection exploded in the second half of the 20th century, the ensuing flood of empirical evidence showed that female animals as diverse as birds, mammals, and insects choose among eager, indiscriminate males [5]. In contrast, mate choice by males was assumed to be confined to odd-ball species with “reversed” sex roles, like seahorses and phalaropes. In accordance with Darwin's ideas (now framed in the language of genetics), both sexual competition among males and female mate choice were generally seen as selecting on male genetic quality [6], and contributing to the spread of “good genes”. Females were expected to choose males based on the quality of males' sexual displays (courtship dances or songs, bright colours, etc.), because males capable of producing an attractive display were assumed to confer both indirect benefits (enhanced offspring fitness) and direct benefits (e.g., low risk of sexually transmitted disease).

## A Reappraisal of Sexual Coevolution

Beginning in the late 1970s, new ideas increasingly challenged both the view of sexual competition as facilitator of viability-enhancing adaptation, and the “typical” sex roles defined by Darwin and Bateman. A corollary of the Darwin-Bateman model of the sex roles is that males indiscriminately seeking additional mating partners will often encounter females that resist mating. The result is sexual conflict.

Sexual conflict takes two distinct forms. The most obvious form, called “interlocus sexual conflict,” occurs when the sexes employ different traits (controlled by different genetic loci) in a struggle over the outcome of an interaction, such as mating. This results in sexually antagonistic coevolution that can lead to a sexual “arms race” [7]. But such struggles can also lead to a more cryptic (and poorly understood) form of conflict called “intralocus sexual conflict,” because the distinct strategies pursued by each sex are manifested as differential selection on the same genes. In other words, selection on one sex may displace the other sex from its phenotypic optimum as a result of the shared genetic basis of homologous traits in the sexes (i.e., intersexual genetic correlation) [8],[9].

Sexual conflict theory was first formalised in 1979 by Geoff Parker [10]. After many years spent observing the mating behaviours of dung flies, Parker realized (and showed theoretically) that a gene that helps males achieve matings will increase in frequency in the population even if the male phenotype that it produces is harmful to females (e.g., because it results in harassment, injury, or excessive mating for females). (Parker assumed that the male-benefit gene was not expressed in females, thus avoiding the complications of intralocus sexual conflict.) Parker showed that such a male-benefit gene could spread, despite the harm that it caused to females, simply because males expressing the male-benefit phenotype would out-compete males lacking that phenotype. By harming their mates, such males may reduce females' life expectancy and lifetime fecundity, and sexual conflict is therefore predicted to be weak or absent in the case of true lifetime monogamy. In the much more commonplace situation where both sexes mate with multiple partners, however, a male's own fitness will depend only on his short-term fertilization gains from each female and the number of females that he can mate with.

Parker's theoretical work led to the startling conclusion that sexual competition by males could have detrimental consequences for mean female fecundity and, therefore, for the viability and growth-rate of populations—a conclusion supported and extended by subsequent theoretical studies [11],[12]. Sexual conflict theory also necessitated a profound re-examination of longstanding concepts. Female mate choice was reinterpreted as a resistance strategy to reduce mating rate or avoid mating with the most harmful males [13]. Conversely, male courtship was then seen as a strategy for exploitation of innate “receiver biases” in the female nervous system [14], benefiting males by increasing their mating success, but potentially harming females in the process.

Following on the heels of these new ideas, accumulating empirical evidence crystallized what had gone largely unnoticed before: mating is often marked by conflict between the sexes, and both sexes often possess “sexually antagonistic” traits that function to coerce or resist the other sex [7]. For example, it was suggested that the colourful spots of male guppies function to exploit the visual cues females use to search for food (colourful fruits) [15]. Numerous studies showed that females often struggle against males, sometimes aided by special morphological or physiological adaptations, while males may possess specialized clasping organs for clinging to reluctant females, or even transfer toxins that function to manipulate females' reproductive physiology [16]–[18]. Thus, whereas Darwin [2] believed that secondary sexual traits like males' clasping organs facilitated sexual cooperation for the mutual benefit of reproduction, many such traits came to be seen as manifestations of sexual conflict [7].

Our understanding of the scope and implications of sexual conflict in evolution is still far from complete. Some researchers believe that sexual conflict theory can illuminate a wide range of questions, such as the evolution of life histories and ageing [19]. Others question the importance of conflict in sexual coevolution [20]. These are exciting times for evolutionary biologists.

## A Reappraisal of the Sex Roles

While sexual coevolution was being re-examined in light of sexual conflict theory, the sex roles were also undergoing a conceptual reappraisal. It was recognized that anisogamy and greater investment in offspring by females do not necessarily favour greater choosiness on the part of females. Even if they invest less per offspring, males can often benefit by being choosy about their mates, for two reasons [21]–[24]. First, mating is often quite costly for males in terms of time, energy, risk, and lost opportunities. Second, females typically vary a great deal in quality, so that a male stands to gain varying amounts of fitness from mating with different females. Empirical work by Darryl Gwynne and others showed that mate choice by males is commonplace [23] and, indeed, that the sex roles can change in response to environmental variables, such as food abundance [25]. Thus, males may often exhibit both competitiveness and choosiness by grappling most vigorously for high-quality females.

In some species, male preferences appear to enhance the fitness of attractive females. For example, when males entice females with nutritious nuptial gifts of glandular secretions or prey items, attractive females (or their offspring) may benefit by receiving more of such gifts [26]. In such species (in contrast to Bateman's [3] findings for *D. melanogaster*), females may be selected to “forage” for additional matings. Nonetheless, the importance of male mate choice has remained controversial because, unlike female mate choice, male preferences typically focus on direct indicators of female fecundity such as body size, and rarely result in the evolution of costly sexual displays in females [27].

## A Role for Male Mate-Choice in Sexual Conflict

Just as sexual conflict theory has clear implications for our interpretation of female mate choice, so too does it necessitate a reappraisal of male mate choice. After all, if we accept that mating is often in the interests of the male but not the female and that traits that enhance males' sexual competitiveness are often harmful for females, then it follows that those females that are most attractive for males may incur the greatest harm. Connecting these dots is simple only in hindsight, of course, but the study by Tristan Long and colleagues in this issue of *PLoS Biology* does just that. Through a series of experiments, Long and colleagues showed, firstly, that *D. melanogaster* males prefer large females. Such a preference has been reported previously [28], and appears to be adaptive for males because larger females carry more eggs and thereby offer more fertilization opportunities. When encountering females of varying body sizes, males therefore direct their attentions preferentially towards the largest females. Secondly, Long et al. showed, for the first time to my knowledge, that these male preferences translate into greater fitness costs for large females than for small ones—a size-dependent manifestation of interlocus sexual conflict. Because large females are more attractive for males, large females suffer more from male harassment [29] and perhaps end up receiving more toxins from male ejaculates [17]. Although larger *D. melanogaster* females still achieve higher mean lifetime fitness than smaller females, the fitness advantage of large body size (and, perhaps, other traits associated with large body size and the genes that give rise to those traits) is diminished as a result of the expression of male preferences. In other words, all else being equal, large females would have done better, relative to smaller females, if males pursued females indiscriminately.

The finding that male mate choice can play a role in sexual conflict forges a link between two hitherto disparate research programs. It also provides a new and compelling reason to care about male mate choice: even if it does not select for exaggerated sexual displays in females, it can potentially impede the evolution of key traits, such as body size, and perhaps contribute to the population-level costs of sexual conflict.

## Where to From Here?

So now we know that males are often choosy and that male–female interactions are often characterized by sexual conflict. We also have reason to believe that male mate choice can sometimes enhance and sometimes diminish the fitness of attractive females. Where do we go from here?

Much more research is needed to understand the nature, importance, and consequences of male preferences, particularly in natural populations, and on longer evolutionary time scales. Since we can no longer justify the assumption that males mate indiscriminately, it is time to integrate male mate choice fully into theoretical and empirical studies of sexual coevolution.

Models of male mate choice [21],[30] have generally ignored the potential for male preferences to affect female fitness, even though such effects can influence selection on male choosiness itself. Long et al. present a simple-population genetic model to illustrate how male mate choice can affect the rate of the spread of a mutation that makes females more fecund as well as more attractive. However, the potentially complex evolutionary dynamics of such systems have yet to be explored.

A variety of interesting questions can be asked. How often do males encounter multiple females simultaneously and have the opportunity to exercise choice? What female traits, besides body size, are targeted by sexual selection? In species where males bear nuptial gifts, females have sometimes evolved signals that enhance their apparent fecundity [31]. Could male preferences sometimes also lead to the evolution of female traits that mask high fecundity? Indeed, what factors determine whether male preferences enhance or diminish the fitness of attractive females? Could male preferences promote the evolution of enhanced defenses against male harm in high-quality females? Most interestingly, could male mate choice impede adaptation to the environment by penalizing the best-adapted females, as suggested by Long and colleagues?

Intriguing questions are also raised by intralocus sexual conflict. This conflict challenges the concept of female mate choice for “good genes,” because high-quality males may sire low-quality daughters [32]. Indeed, mutual mate choice may be expected to lead to pairing between males and females of similar quality [24], a situation that, under the assumptions of classic sexual selection models, would enhance the fitness advantages of high-quality individuals of both sexes [33]. Given intralocus sexual conflict, however, such assortative mating for quality may lead to the paradoxical situation whereby the highest quality individuals tend to produce the lowest quality offspring [32]. Intralocus sexual conflict, therefore, appears to negate the indirect benefits of female mate choice and perhaps favours indiscriminate mating by females. The situation is somewhat different from the male perspective, however. Because male preferences generally focus on phenotypic indicators of direct benefits, such as high fecundity, rather than good genes [27],[28], choosiness is likely to be advantageous for males, despite intralocus sexual conflict, because such preferences will result in increased offspring number for choosy males. Intralocus sexual conflict therefore predicts that male mate choice will be more widespread relative to female mate choice than is commonly assumed. Moreover, if male and female preferences are genetically correlated, then selection on preferences in one sex may cause correlated evolution of preferences in the other sex. An intersexual genetic correlation for preference is not improbable, given that the sensory systems of the sexes are typically similar and controlled by a shared genetic machinery. The role of intralocus sexual conflict in sexual coevolution remains poorly understood.

In short, if we could resurrect Charles Darwin, I believe he would be very surprised and perhaps even deeply troubled by recent developments in evolutionary biology. I'll wager that the future will surprise us all.
